# Shear bond strength of the ceramic veneer to additively manufactured titanium

**DOI:** 10.1002/cre2.820

**Published:** 2023-12-03

**Authors:** P. Svanborg, H. H. Le, S. J. Sigurðardóttir, S. Barkarmo

**Affiliations:** ^1^ Department of Prosthodontics/Dental Materials Science, Institute of Odontology, The Sahlgrenska Academy University of Gothenburg Göteborg Sweden

**Keywords:** dental alloys, dental porcelain, dental prosthesis, titanium

## Abstract

**Objectives:**

The objective of this in vitro study was to evaluate the shear bond strength between the ceramic veneer and additively manufactured titanium with different surface treatments, and to compare with milled titanium. Also, to characterize the surface and the presence of an α‐case layer of additively manufactured and milled titanium.

**Material and Methods:**

Sixty additively manufactured titanium grade 23, and 20 milled titanium grade 4 cylindrical specimens were divided into four groups based on surface treatments, air‐particle abrasion and grinding. After ceramic veneering half of each group were thermocycled. The bond strength was analyzed using a shear bond strength test. The surfaces were analyzed using interferometry and scanning electron microscopy.

**Results:**

The grinding procedure and air‐particle abrading pressure had no significant effect on the shear bond strength (*p* = .264 and *p* = .344). Thermocycling showed a tendency towards an effect but not significant (*p* = .052). The group with the highest air‐abrading pressure showed the highest surface roughness. No presence of an α‐case layer was detected in any of the groups.

**Conclusion:**

Additively manufactured titanium grade 23 may be veneered with ceramics without prior grinding of the surfaces.

## 1 INTRODUCTION

Commercially pure (CP) titanium (Ti) has been used in prosthetic dentistry since the 1980's. In recent years Ti alloys such as TiAl6V4, TiAl6V4 ELI, and TAN have been introduced. CP‐Ti and Ti alloys have a high melting point (1668°C) and must be cast in a special casting machine with an argon atmosphere. Also, when the molten metal comes in contact with the investment a reaction occurs which creates a surface layer called α‐case. Depending on the type of investment material the thickness of the α‐case layer may be between 14 and 80 µm (Guilin et al., 2007). The α‐case layer, which is rich in oxygen, is hard and brittle and may contain microcracks (Chan et al., 2008). Therefore, it must be removed by grinding before ceramic veneering (Muiruri et al., 2020; Shen, 2013). Since Ti has a coefficient of thermal expansion of about 8–10 and undergoes an allotropic transformation at 882°C, special low‐fusing ceramic veneering materials with sintering temperatures below 800°C must be used (Shen, 2013).

Titanium forms a Ti oxide layer (TiO_2_) in contact with oxygen, approximately 5–10 nm thick, the passivation oxide layer enhances the anticorrosive behavior and is also important for the bond between Ti and the ceramic veneering material (Kasemo BL, 1985; Kasemo, 1983). The thickness of the oxide layer is not significantly affected by oxidation heat treatment up to 750°C. However, with higher temperatures, up to 1000°C, the oxide layer will grow to a thickness of 1μm. Such a thick oxide layer will significantly decrease the adherence of the oxide to both CP‐Ti and TiAl6V4 which will affect the bond strength to the ceramic veneering material (Adachi et al., 1990).

The bond strength between cast CP‐Ti grade 2 and two different veneering ceramic materials with and without surface modification was studied using the three‐point bending test. The mean bond strength values ranged from 17.2 to 24.9 MPa (Özcan & Uysal, 2005). In another study milled CP‐Ti grade 2 had a bond strength above 28 MPa with air‐particle abrasion only, and above 35 MPa with a Au sputter coating (Lin & Huang, 2014). The bond strength of TiAl6V4 and a ceramic veneering material was above 32 MPa with air‐particle abrasion and was improved further using a borate bonder, up to 49 MPa (Zhao et al., 2016). To increase the bond strength of the ceramic veneering material to Ti a bonder can be applied, good results can be attained by using a Au bonder or coatings of Au or Ti nitride (Al Hussaini & Al Wazzan, 2005; Park et al., 2009; Tholey et al., 2007). Air‐particle abrasion with 250 µm aluminum oxide particles with 0.2 to 0.3 MPa pressure for 10 s on a polished Ti surface will increase the roughness from 0.25 to 1.49 µm on CP‐Ti grade 2 and from 0.9 to 1.02 µm on TiAl6V4 (Mohsen, 2012). Thermocycling is used to simulate clinical use in vitro, some studies have reported a weakening of the bond strength (Mohsen, 2012; Sendão et al., 2015; Vasquez et al., 2009) and others found no significant difference (Tróia et al., 2003; Vásquez et al., 2009).

A high rate of ceramic chip off fractures has been reported in clinical follow‐up studies on Ti restorations. Kaus et al. followed 84 restorations with 125 veneers for 3 years. Ceramic fractures were reported for 25.9% of the veneers and mainly in the multi‐unit FDPs (Kaus et al., 1996). In another study, 41 single crowns followed for 6 years reported ceramic fractures in 27% of the crowns. The high fracture rate may in part be explained by a uniform coping design and thus an insufficient support for the veneering ceramic material (Hey et al., 2014). In contrast, Milleding et al. followed 40 single crowns for 2 years with only one ceramic fracture (Milleding et al., 1998) and Nilson et al. reported two fractures in 44 crowns after 26–30 months (Nilson et al., 1994).

Ti restorations are also produced by using subtractive techniques, eg. milling and spark erosion (Andersson et al., 1996). Additionally, additive manufacturing (AM) techniques such as electron beam melting (EBM), laser beam melting (LBM), and selective laser sintering (SLS) are used (Papia et al., 2017; Svanborg et al., 2018). In an earlier study, evaluating the fit of implant‐supported CoCr and Ti frameworks before and after ceramic veneering, the veneering of the AM Ti frameworks was accompanied with complications such as air bubbles and cracks in the ceramic veneer (Svanborg et al., 2018).

The ceramic bond strength for cast, milled, and AM Ti has been studied by İşeri̇ et al. who reported shear bond strength values for SLS 32.2 MPa, milled 18.9 MPa, and cast Ti 28.6 MPa (Iseri et al., 2011). Papia et al. also studied the shear bond strength and the effect of passivation (P) and no‐passivation (NP) for cast Ti P 24.1, NP 23.3, milled Ti P 18.5, NP 21.3, and EBM Ti P 22.5, NP 25.0 (Papia et al., 2017). The relationship between AM Ti, surface treatment, α‐case layer, and ceramic‐alloy bond strength needs to be clarified.

The purpose of this in vitro study was to evaluate the shear bond strength between the ceramic veneer and additively manufactured titanium with different surface treatments, and to compare with milled titanium. Also, to characterize the surface and the presence of an α‐case layer of additively manufactured and milled titanium.

The hypotheses are that there is no difference in shear bond strength between the ceramic veneer and the two production methods with the different surface treatments. And, that there is no presence of an α‐case layer with either of the two methods.

## 2 MATERIALS AND METHODS

Computer aided design (CAD) software was used (Fusion 360; Autocad) to create a standard tessellation language file (STL) of a cylindrical specimen of 4.9x5mm (diameter/length), which was exported and sent to a production center (Atlantis Suprastructures; Dentsply Implants NV, Hasselt, Belgium). Using a Renishaw AM250 machine and titanium alloy powder (TiAl6V4 ELI), 60 AM Ti specimens were fabricated. Also, a control group of 20 milled CP Ti grade 4 specimens was fabricated.

### 2.1 Surface treatments

**Figure 1:**
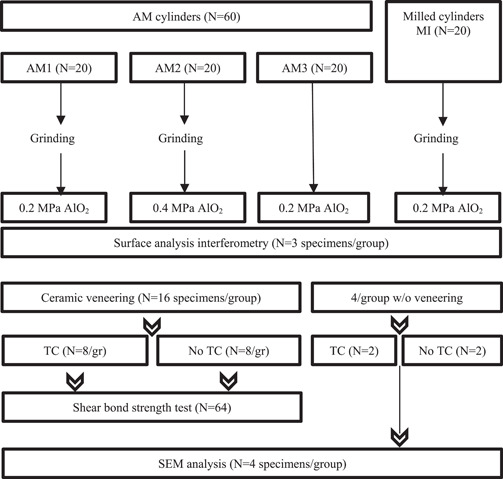
Flow‐chart of groups, surface treatments and analyses.

The specimens were given different pre‐treatments, 20 AM (AM1) and 20 milled specimens (MI) were grinded using 120, 500, and 1000 grit waterproof silicon carbide paper under water cooling (RotoForce‐4; Struers), air‐particle abraded with 110 µm AlO_2_ at 0.2 MPa (Basic classic; Renfert) for 10 s and left to rest for 15 min before steam cleaning (Supersteam; Reitel) for 60 s. The second group of 20 AM (AM2) specimens was grinded using the same protocol and air‐particle abraded with 110 µm AlO_2_ at 0.4 MPa for 10 s and left to rest for 15 min before steam cleaning. The third group of 20 AM (AM3) specimens was only air‐particle abraded with 110 µm AlO_2_ at 0.2 MPa for 10 s and left to rest for 15 min before steam cleaning (Figure 1).

### 2.2 Ceramic veneering

16 specimens from each group were veneered with ceramics using a calibrated firing furnace (Austromat 300; Dekema) by two of the authors (HH.L., SJ.S.). Bonder and opaque paste were applied and fired according to the manufacturer's instructions (Titan Keramik paste bonder/Titan Keramik Opaque A3; VITA Zahnfabrik). A silicone mold was used to facilitate the application of dentine ceramic according to the required measurements (TitanKeramik Dentine A3; VITA). The build‐up dentine ceramics was applied and fired twice due to shrinking, approximately two mm. A stone was used to grind any abundance of ceramic and ceramic fluid at the transition between ceramic and titanium.

### 2.3 Thermocycling

Half of the veneered specimens from each group (*n* = 8) were subjected to thermocycling (Kübler 901; Kübler). The specimens were placed in 5–55°C water for 30 s in each temperature, with a 5 s transfer. The specimens were subjected to 5000 thermocycles, and the groups were given the suffix TC.

### 2.4 Surface analysis

The surface roughness was analyzed using interferometry (White light interferometer, Smart WLI‐extended optical 3D surface Measuring system; GBS) on three specimens from each group before ceramic veneering. Three measurements were made on each specimen. The arithmetic mean height (S_a_), developed interfacial area ratio (S_dr_), and density of summits (S_ds_) parameters were chosen for analysis (Wennerberg & Albrektsson, 2000).

Another four specimens from each group were analyzed for presence of an α‐case layer. The specimens were sectioned (Secotom‐60; Struers) and mounted by using resin (ClaroCit; Struers) in a purpose‐built brass holder. Grinding and polishing was performed using a semi‐automatic machine (Planpol; Struers). All specimens were etched for 2 min with a solution containing 100 ml distilled H_2_O, 3 ml concentrated HCl, and 6 ml concentrated HNO_3_. The sample surfaces were rinsed in ethanol and air blown dry before coating with a AU85Pt15 alloy, approximately 5 nm in thickness. Scanning electron microscopy (SEM) was performed at 10 keV (TM4000plus; Hitachi). The specimens were imaged at 500x, 1000x, and 2000x magnification.

### 2.5 Shear bond test

The veneered specimens (16/group) were placed in a universal testing machine (Lloyd LRX; Lloyd Instruments) for the shear bond strength test in a cylindrical “guillotine.” The shear load was set at 0.5 mm/s and applied at the transition between ceramic and titanium. The final load at fracture was registered in N. The average shear bond strength (MPa) was calculated by dividing the load at fracture with the ceramic bond area (mm^2^).

After the shear bond test all the specimens were analyzed using a microscope (Nikon SMZ800; Nikon) for type of fracture. Adhesive, cohesive, or mixed fracture was defined by the amount of ceramic left on the surface. If the majority of the surface was exposed Ti the fracture was adhesive, if the majority was ceramic, it was cohesive. An equal amount of exposed Ti and ceramic was defined as a mixed fracture.

### 2.6 Statistical analysis

The data were analyzed using a statistical software program (IBM SPSS Statistics, v26; IBM). The independent samples Mann‐Whitney U test was used to analyze the effect of grinding, air‐particle abrading pressure, and thermocycling on shear bond strength stepwise (*α* = .05).

## 3 RESULTS

**Figure 2:**
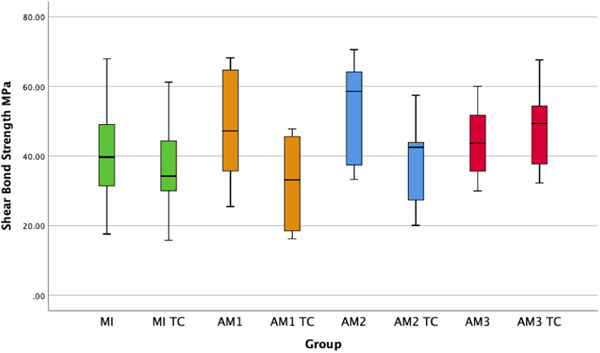
Box‐plot of shear bond strength.

The results from the shear bond strength test are presented in Figure 2. The grinding procedure and air‐particle abrading pressure had no significant effect on the shear bond strength (*p* = .264 and *p* = .344). Thermocycling showed a tendency towards an effect but not significant (*p* = .052). When AM3 was excluded from the analysis, thermocycling significantly lowered the shear bond strength for the grinded groups (*p* = .015). The surface roughness parameter S_a_ was higher for AM2 (0.4 MPa pressure) compared to the other groups (Table 1).

**Table 1:** Surface roughness measured on three specimens per group.

	**Mean (Std. deviation)**		
**Group**	**S**_**a**_ µm	**S**_**ds**_ %	**S**_**dr**_ 1/µm^2^
MI	.89 (0.05)	34.7 (1.9)	.204 (0.005)
AM1	.91 (0.03)	33.7 (1.8)	.198 (0.004)
AM2	1.3 (0.04)	41.3 (2.1)	.196 (0.004)
AM3	.99 (0.04)	34.4 (1.8)	.187 (0.004)

**Figure 3:**
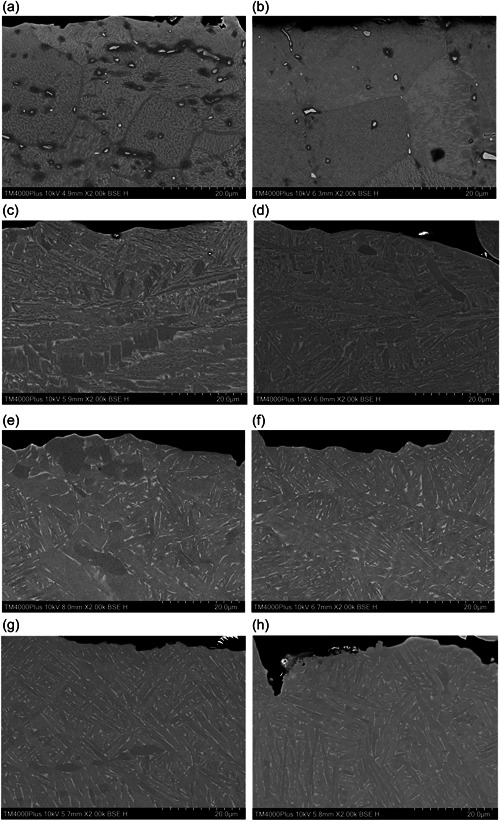
Examples of SEM images of the specimens, magnification x2000 (a) MI, (b) MI TC (c) AM1, (d) AM1 TC, (e) AM2, (f) AM2 TC, (g) AM3, (h) AM3 TC.

The microstructural analysis showed no evidence of an α‐case layer for any of the groups. The MI group presented a different microstructure compared with the AM groups. The grain structure in the MI group had equiaxed grains. The AM groups had a basket weave pattern of lamellar grains (Figure 3a‐h).

The type of fracture for the groups MI, AM1, and AM2 was a mix of adhesive, cohesive, and mixed fractures before thermocycling. After thermocycling the type of fracture changed to a majority of adhesive fractures. No change was recorded for AM3 (Table 2).

**Table 2:** Fracture classification after shear bond strength test.

	Type of fracture		
Group	Adhesive	Cohesive	Mixed
MI	1	3	4
MI TC	7	1	
AM1	6	2	
AM1 TC	6		2
AM2	4	1	3
AM2 TC	8		
AM3	7		1
AM3 TC	7		1

## 4 DISCUSSION

The hypothesis that there would be no difference in shear bond strength regardless of production method or surface treatment had to be rejected. However, the hypothesis that there would be no presence of an α‐case layer with either production method was accepted.

The production method, additive or subtractive manufacturing, affected the microstructure of the specimens. The MI specimens presented equiaxed grains and the AM specimens had lamellar shaped grains. Other studies on production methods have found the same type of microstructures for AM and equiaxed or lamellar for MI techniques depending on heat treatment (Attanasio et al., 2013; Murr et al., 2009; Xiao et al., 2019). The microstructure of titanium alloys can also affect their mechanical properties. Alloys with a lamellar microstructure are characterized by a high fracture toughness and lower strength and ductility, an equiaxed microstructure has a higher strength and ductility, and a lower fracture toughness. The grain size is also a factor, coarse grains are more ductile compared with fine grains (Kang & Yang, 2019; Wysocki et al., 2017). The differences could also be explained by the composition of the materials, the MI group was made of CP Ti grade 4 and the AM groups of Ti grade 23 (TiAl6V4 ELI). The reason for the choice of materials was based on the materials and production techniques used by Dentsply Sirona at the time, to reflect the choices available for the dental laboratory. A small difference in coefficient of thermal expansion between the materials could result in different amount of residual stress in the Ti‐ceramic interface (Vásquez et al., 2009).

The surface roughness values for the three roughness parameters were comparable between the two groups MI and AM1, implying that the manufacturing method may not be a significant factor in determining roughness. However, other factors such as differences in alloy composition and post‐processing may be important. Nevertheless, the air‐particle abrading pressure affected the surface roughness. A higher pressure resulted in a higher surface roughness. This confirms the results from earlier studies. Mohsen showed that a pressure of 0.2–0.3 MPa increased the surface roughness of TiAl6V4 to comparable values with AM2 which was air‐particle abraded with 0.4 MPa (Mohsen, 2012). Golebiowski et al. found that an increase in pressure from 0.2 to 0.4 MPa increased the Ra value from 0.58 to 1.03 µm (Golebiowski et al., 2015). Air‐particle abrasion must be performed before ceramic veneering to clean the surface and produce a rough surface with micro‐mechanical retention for the ceramic (Reyes et al., 2001; Solomon, 1980; Taira et al., 1990). But according to the results from this study grinding can be excluded for AM titanium. With the development of digital techniques such as CAD software programs and additive manufacturing, the restorations produced with AM today are of good quality and can be processed without corrective grinding of the surface. AM titanium restorations may therefore be produced with individualized micro and macro surface designs to utilize the possibilities with AM technology.

An interesting find in this study was that the results for thermocycling presented an heterogenous effect between the groups which was not expected. For the AM specimens that were only air‐particle abraded the thermocycling improved the result in shear bond strength and the type of fractures between Ti and the ceramic were not affected. For the other groups thermocycling significantly lowered the bond strength, which could be an indication that clinical use could negatively affect the bond and increase the risk of clinical fractures (Vásquez et al., 2009) More analyses or tests would be needed to explain or exclude the possibility of a random error. Also, the type of fracture changed to a majority of adhesive fractures. The fracture in shear bond strength tests usually transpire in the weakest part of the assembly. Adhesive fractures occur through the oxide layer. A weaker oxide layer could be interpreted as an effect of thermocycling, which lowers the bond strength. However, this effect was not seen in the group which was only air‐particle abraded.

The shear bond strengths presented here are higher compared with earlier studies on shear bond strength to titanium (Golebiowski et al., 2015; Iseri et al., 2011; Papia et al., 2017). According to ISO9693 the bond strength for metal‐ceramics should be analyzed using the three‐point bending test (ISO, 2012). However, there are limitations to that method. It is difficult to produce specimens with a distinct 90° edge between the ceramic and metal. In many cases, the ceramic has a larger contact surface to the metal than specified in the standard which may affect the calculations. Also, the specimens should be ground before air‐abrading and ceramic veneering. To include a group without grinding, the shear bond strength test was chosen.

The statistical methods used in this study were chosen to compare the groups stepwise. A more sophisticated method, perhaps a regression analysis, could have been more suitable to find interactions between different factors. But with the small sample sizes the test would have been sensitive to outliers. The results in this study are based on a limited laboratory experiment. Therefore, since these materials are already used in a clinical setting retrospective clinical follow‐up studies could be performed to confirm these results.

In conclusion, additively manufactured titanium grade 23 may be veneered with ceramics without prior grinding of the surfaces.

## AUTHOR CONTRIBUTIONS

Per Svanborg: First author, conceptualization, methodology, investigation, analysis, writing original draft, given final approval of the version to be published, agreed to be accountable. Ho Henry Le: Conceptualization, methodology, investigation, analysis, writing original draft, given final approval of the version to be published, agreed to be accountable. Sigrún Sigurðardóttir: Conceptualization, methodology, investigation, analysis, writing original draft, given final approval of the version to be published, agreed to be accountable. Sargon Barkarmo: Conceptualization, methodology, analysis, supervision revising draft critically, given final approval of the version to be published, agreed to be accountable.

## CONFLICTS OF INTEREST STATEMENT

Dr. Svanborg has nothing to disclose. Mr. Le has nothing to disclose. Ms. Sigurðardóttir has nothing to disclose. Dr. Barkarmo has nothing to disclose.

## Data Availability

Research data are not shared.
